# The adventures of psychiatric reform in Greece: 1999–2019

**DOI:** 10.1192/bji.2019.30

**Published:** 2020-05

**Authors:** Michael G. Madianos

**Affiliations:** Emeritus Professor of General and Social Psychiatry, School of Health Sciences, Department of Mental Health and Behavioural Sciences, University of Athens, Greece. Email: madianos@nurs.uoa.gr

**Keywords:** Greece, psychiatry, reforms

## Abstract

Psychiatric care in Greece has a long history of traditional in-patient treatment in large public institutions (the public asylum period), which lasted until 1983. European Economic Community (EEC) intervention in 1984 marked a transitional period (1984–1989) characterised by the beginning of the transformation of the mental healthcare system. The current reform era started in 1999 and has included new mental health law, the closure of six public mental hospitals and the establishment of several sectorised mental health services.

The historical evolution of psychiatric care in Greece can be divided into three extended periods. First, the asylum period, starting with the founding of the first public mental hospitals in the country in the late 19th century. They were the prevalent type of care in the public sector until the early 1980s. This was followed by a transitional period that resulted largely from the European Economic Community's (EEC's) intervention, which led to reform of the mental healthcare system 3 years after Greece had become a member state in 1981. The launching of a National Mental Health Plan in 1997 and the introduction of new legislation in May 1999 (Law 2716/99) marked the beginning of the third period of psychiatric reform.

## The epoch of public asylums (to 1983)

The first psychiatric hospital in the country was founded in 1834 in Corfu during the British occupation of the Ionian Islands. Three decades later, in 1862 mental health legislation was passed largely influenced by the respective French legislation of 1838.^1^ In the Athens area, Dromokaition Mental Hospital was founded in 1887, followed by Eginition University Hospital 12 years later. It was not until the mid-1930s that the biggest state mental hospital of Athens, Dafni Psychiatric Hospital, was established, which, with eight other regional psychiatric hospitals, also provided in-patient psychiatric care.

All of these facilities catered for patients suffering mainly from psychoses and organic brain syndromes. Most hospitals were relatively small until the first half of the 20th century. In subsequent decades, owing to urbanisation and profound socioeconomic and political changes (including the Second World War, which led to the German Occupation and civil war), their patient population dramatically increased, and some small institutions were transformed into large asylums.^2^

Following the fall of the military dictatorship in Greece in 1974, the sociopolitical atmosphere changed, as reflected in the democratisation of civil liberties, and in education, work conditions and private healthcare insurance, but these changes did not have much impact on services for mental health conditions.

The psychiatric care delivery system remained based largely on nine overcrowded asylums, which were inadequately staffed and contained a total of 9260 beds; there were also a number of private psychiatric clinics.^3^

The mental healthcare system in the early 1980s was mainly characterised by the centralisation of psychiatric services. There was a lack of any community-based structure, and an uneven distribution of psychiatrists per number of beds. There was also unequal staffing between facilities, poor quality of treatment and inadequate distribution of funds.^4^ In terms of length of stay, the majority of in-patients were long-stay residents, most exceeding 365 days.^2,3^ Popular concepts about mental illness reflected traditional beliefs and projected stigmatising attitudes.^5^

While Greece was facing this inadequate mental healthcare system, community psychiatry and the transformation of psychiatric services had already been introduced in several north-western European countries, with the closure of psychiatric hospitals and the deinstitutionalisation of numerous long-stay patients.^6^ In southern Europe, there was little change, with the exception of the Italian psychiatric movement initiated by Franco Basaglia (a professor of psychiatry and neurology) and his colleagues. They believed that much of the behaviour associated with ‘madness’ resulted from institutional confinement, a view that echoed the ideology of May 1968 (when radical psychiatry/antipsychiatry opinions were reflected in the general civil unrest), and their actions led to the dismantling of mental hospitals throughout Italy in the late 1970s. In Greece, in contrast, during the late 1970s only a few innovative pilot mental health projects were implemented by inspired mental health professionals.^7^

## The transitional period (1984–1998)

In 1983 the socialist government introduced the new National Health System in Greece (Law 1397/83). Reform in mental healthcare had become imperative and Article 21 provided the basis for the decentralisation of mental healthcare.^4^ During the same period, the ‘Leros asylum scandal’ broke, provoking strong negative European reactions.^8,9^ Leros, which is part of a Dodecanese island cluster in the Aegean Sea, had become infamous because of its large psychiatric asylum, housing over 2000 inmates in tragic ‘treatment’ conditions. This asylum was established in 1957 as an ‘agricultural colony of psychopathic persons’. For many years it accepted long-stay in-patients, who had been abandoned by their relatives, from all other state mental hospitals across the country. It had very few trained staff. By 1982, when the transfer of patients stopped, patient numbers had increased disproportionately compared with available medical and nursing staff^10^ and as recently as 1989 there were just two psychiatrists and one general medical practitioner for 1138 patients. As a result of international pressure and the risk of condemnation of Greece by the European Tribune in Strasbourg, the EEC offered a grant of 120 million ECU under Regulation 815/84 Programme B, for Greece to undertake major reform of its psychiatric services. The Greek government undertook revision of the mental healthcare system by developing a 5-year plan (1984–89). This had to be extended owing to inadequate disbursement of the donated funds, caused mainly by bureaucracy and a lack of experience. By 1990, only 24% of the EEC funds had been used for the purpose for which they had been donated, so the programme was further extended to 1995.^3,11^

The strategic objectives of this plan were as follows. First, the evacuation of the Leros asylum (the first 250 ex-patients had already been sheltered in local community hostels). Second, the decentralisation of mental health services in the country and the deinstitutionalisation of long-stay patients. Third, the improvement of living conditions in all public mental hospitals.^8,10^ A total of 140 new innovative infrastructures and intervention projects were developed during the 1984–1995 period, and between them they almost totally absorbed the EEC Regulation 815/84 funds. In 1989, the Hellenic Psychiatric Association established a task force which visited the asylum on Leros and prepared a report with recommendations that were sent to the Greek Ministry of Health and the relevant EEC directorate.^12^

## The reform era (from 1999)

A 10-year National Mental Health plan, ‘Psychargos’, was initiated and approved by the European Union (EU) in 1997 and was funded by 700 million euros. Its objectives mainly focused on the deinstitutionalisation of the remaining long-stay in-patients and the closure of the public mental hospitals. It also aimed to introduce mental health legislation that would secure the sectorisation of the mental healthcare system (having the same team responsible for in-patient and out-patient care in a particular catchment area), the further development of community-based mental health and rehabilitation services, the launching of psychiatric departments in general hospitals, as well as providing specialised services for children, adolescents and the elderly. Finally, detailed guarantees and procedures for the protection of patients' rights were established.^9^ In line with the new Mental Health Plan, legislation was introduced in 1999 (Law 2716/99) that constituted a thorough and comprehensive policy on mental health. It focused on the sectorisation of the country, the protection of the human rights of the mentally ill and emphasised the importance of developing a network of primary mental health services that covered the country.^3,4,13^ In 2002, the whole country was sectorised, with a revision in 2015 into 7 health regions and 37 sectors.

‘Psychargos I’ lasted from 1997 until 2001 and led to an improvement in infrastructure and the transfer of long-stay patients to community-based alternative structures, as well as the training of personnel. ‘Psychargos II’ lasted from 2001 to 2010 and was marked by the closure of six public mental hospitals.^3^ Hundreds of inmates were sheltered in places of their origin in mainland Greece by various agencies, university departments, non-governmental organisations, the Hellenic Centre for Mental Health and others.

The remaining three hospitals have been serving as acute care units linked to consecutive sectors, with 419 short-stay beds in Athens and 237 in Thessaloniki. Under the ‘Psychargos’ programmes, 23 new community mental health centres (CMHCs), 53 out-patient clinics and 28 psychiatric departments in general hospitals were instituted.

An overall picture of the mental healthcare delivery system in Greece is shown in Table 1. It is obvious that there has been a dramatic increase in the number of units, beds and places in all types of services between 1984 and 2019. It should be noted that there are still many psychiatric beds in general hospitals. The currently available 3847 alternative residential beds indicated in the table cater to the needs of deinstitutionalised patients.

**Table 1:** Mental healthcare delivery in Greece (1984–2019)

	1984^a^	1990	1999	2010	2019
Beds in general hospitals, *n*	36	281	407	480	568
Community mental health centres,^b^ *n*	7	19	32	34	49
Mental health services for children, *n*	8	22	28	28	34
Out-patient departments in general hospitals, *n*	20	43	50	53	53
Mobile units	2	6	17	20	20
Places in day care centres, *n*	55	381	390	615	725
Social cooperatives/enterprises	–	–	–	18	24
Beds in residential alternatives, *n*	15	522	1962	3057	3847
Alzheimer's centres and hostels, *n*	–	–	4	6	13
Autism spectrum specialist centres and hostels, *n*	2	3	5	10	17
Drug and alcohol detox programmes, *n*	5	18	53	92	105

a. 1984 is taken as the baseline year because of EEC Regulation 815/84.

b. Nine of the current community mental health centres also provide specialised services for children and adolescents.

## Conclusions

In Greece, psychiatric reform has been characterised by a slow process and by retrogressive steps; it has not been motivated by public opinion (either professionals or citizens). The main target of reform has been the deinstitutionalisation of thousands of asylum patients and their transfer to community-based alternative structures. Despite the fact that many changes have been achieved by the reform plan, there still exist some weaknesses to be addressed. For example, numerous infrastructures have developed unevenly; the CMHCs are not operating on a 24-hour basis and their effectiveness has not been assessed. Community mental health ideology and social psychiatry principles have not been part of the training of personnel and there has been no community participation in intervention programmes. On the other hand, some positive initiatives have developed. These include an anti-stigma movement and the formation throughout Greece of societies comprising the families of people with mental illness, as well as Greek representatives of the Hearing Voices network, which was founded there in 2010. In conclusion, as it has become obvious, that the psychiatric reform is still making progress in several fields despite some inadequacies and constraints. Still, much remains to be done in the areas of prevention policies, primary care training and evaluation research through the monitoring and restructuring of the National Mental Health Plan.
